# Testing the Aquatic Toxicity of 2D Few-Layer Graphene Inks Using Rainbow Trout (*Oncorhynchus mykiss*): In Vivo and In Vitro Approaches to Support an SSbD Assessment

**DOI:** 10.3390/toxics12020097

**Published:** 2024-01-23

**Authors:** Gregorio Molés, Mona Connolly, Ana Valdehita, Gerardo Pulido-Reyes, Maria L. Fernandez-Cruz, Emmanuel Flahaut, José M. Navas

**Affiliations:** 1 Department of Environment and Agronomy, Instituto Nacional de Investigación y Tecnología Agraria y Alimentaria (INIA), Consejo Superior de Investigaciones Científicas (CSIC), Ctra. de La Coruña, km 7.5, 28040 Madrid, Spain; gmoles@ciimar.up.pt (G.M.); connolly.mona@inia.csic.es (M.C.); ana.valdehita@inia.csic.es (A.V.); gerardo.pulido@uam.es (G.P.-R.); fcruz@inia.csic.es (M.L.F.-C.); 2 Centro Interdisciplinar de Investigação Marinha e Ambiental (CIIMAR), Terminal de Cruzeiros do Porto de Leixões, 4450-208 Matosinhos, Portugal; 3 Centre Inter-Universitaire de Recherche et D’Ingénierie en Matériaux (CIRIMAT), Centre National de la Recherche Scientifique (CNRS), 16 Av Edouard Belin, 31400 Toulouse, France; emmanuel.flahaut@univ-tlse3.fr

**Keywords:** graphene, aquatic ecotoxicity, fish, OECD test guidelines, EROD, BFCOD

## Abstract

Graphene-based conductive inks offer attractive possibilities in many printing technology applications. Often, these inks contain a mixture of compounds, such as solvents and stabilizers. For the safe(r) and sustainable use of such materials in products, potentially hazardous components must be identified and considered in the design stage. In this study, the hazards of few-layer graphene (FLG)-based ink formulations were tested in fish using in vitro (RTL-W1 cell line) and in vivo aquatic ecotoxicity tests (OECD TG 203). Five ink formulations were produced using different processing steps, containing varying amounts of solvents and stabilizers, with the end products formulated either in aqueous solutions or in powder form. The FLG ink formulations with the highest contents of the stabilizer sodium deoxycholate showed greater in vitro cytotoxic effects, but they did not provoke mortality in juvenile rainbow trout. However, exposure led to increased activities of the cytochrome P450 1a (Cyp1a) and Cyp3a enzymes in the liver, which play an essential role in the detoxification of xenobiotics, suggesting that any effects will be enhanced by the presence of the stabilizers. These results highlight the importance of an SSbD approach together with the use of appropriate testing tools and strategies. By incorporating additional processing steps to remove identified cytotoxic residual solvents and stabilizers, the hazard profile of the FLG inks improved, demonstrating that, by following the principles of the European Commission’s safe(r) and sustainable by design (SSbD) framework, one can contribute to the safe(r) and sustainable use of functional and advanced 2D materials in products.

## 1. Introduction

Graphene, an allotrope of carbon, is defined as a single layer of graphite with carbon atoms arranged in a two-dimensional honeycomb lattice nanostructure [1]. Graphene represents an attractive material for a wide range of technical applications in a variety of fields, including the chemical industry, healthcare, electronic devices, and biomedical research [2]. Graphene nanoforms can exist as monolayer graphene, few-layer graphene (FLG), graphene oxide (GO), reduced graphene oxide (rGO), graphene nanoflakes, nanosheets, nanoribbons, etc. The development of conductive inks using graphene has expanded the possibilities for its application in printed flexible electronics, transistors, sensors, antennas, wearable electronics, smart packaging, and clothing. Graphene inks, which can be described as a dispersion of graphene flakes in solvents, can be used for the formulation of functional and smart paints and coatings, textile finishing, and/or solution-processed polymer compounding to produce functional composite materials [3,4]. These inks, when incorporated into materials, can provide mechanical strength, flexibility, and thermal/electrical conductivity and can be easily applied via spraying, screen-printing, inkjet-printing, and doctor-blading techniques [5]. The use of graphene also offers other advantages over traditional conductive aqueous inks, which contain high concentrations of hazardous metals, and even metal-nanoparticle-based inks, which require high (i.e., up to 80%) material loadings [6,7].

However, it is important to be aware that with the emergence of these new technologies and functional materials there must also be considerations regarding their safe(r) and sustainable development. There is a responsibility to ensure that the design, development, production, and use of new technologies do not impact negatively on human health or the environment at any point through the product lifecycle. In fact, this is in line with the safe(r) and sustainable by design (SSbD) principles that should already be applied to a substance at the design stage to ensure safety, and it is the fundamental principle upon which the European Commission’s recently published recommendation for an SSbD framework was built [8]. According to this framework, the potential environmental impact following emission in water bodies must be assessed, including hazards to aquatic organisms. Graphene and its specific nanoforms are subject to regulation within the European Union [9], and as these materials can exist in many nanoforms, with different properties and functionalizations, hazard assessment on a case-by-case basis may be needed. A recent review of the available graphene-based materials’ ecotoxicity data suggests that certain nanoforms can elicit acute toxic effects on aquatic organisms (e.g., algae and *Daphnia* spp.); however, there are insufficient data to enable concrete conclusions to be drawn concerning the aquatic hazard classification following acute exposure [10]. For example, in the case of FLG, there are only a limited number of studies available, with no mortality reported in fish exposed to 0.2 mg/L [11], and no mortality in zebrafish embryos (0.075 mg/L) [12], whereas EC_50_ values of 10 mg/L were reported in tests when the crustacean *Daphnia magna* was exposed to FLG [13]. Also, the available data suffer from deficiencies/inconsistencies due to the lack of use of standardized approaches (e.g., standardized Organization for Economic Co-operation and Development (OECD) test guidelines (TGs)), so the need for additional considerations when applying standardized tests to graphene materials has been highlighted [10]. The use of new approach methodologies (NAMs) such as in vitro fish-cell-culture-based toxicological testing can also serve as a non-animal alternative and predictive tool for hazard assessment. Such methods will aid in allowing screening for effects directly related to a material’s properties by increasing the ability to test an array of materials with slight modifications in properties, thus allowing for the deduction of potential direct property–effect relationships. The extent to which such approaches can facilitate the prediction of acute toxicity of FLG or serve as a screening approach within an SSbD framework has yet to be fully realized and explored.

Therefore, the objective of this study was to assess the acute aquatic toxicity of a commercial FLG ink product in various formulations and following reprocessing using an in vitro (RTL-W1 cell line) approach, along with a standardized test for acute toxicity assessment in fish (OECD, TG 203). The information generated served to fill an information gap on the acute toxicity of FLG to fish, while also allowing us to compare the influence of different processing methods on the effects (i.e., the graphene ink hazard profile), to support an SSbD approach to meet regulatory needs and foster innovation. In this sense, the present paper not only provides additional data about the toxicity of graphene but also explores the applicability of current and emerging hazard assessment tools in technological development.

## 2. Materials and Methods

### 2.1. Nanomaterials and Stock Dispersions

Few-layer graphene (FLG) inks in aqueous dispersion and powder forms were provided by BeDimensional, a spin-off company of the graphene labs located at the Instituto Italiano di Tecnologia (IIT) in Genoa, Italy. Two different sets of products and substances, which received the general names of FLG A and FLG B, were tested. The aqueous dispersion of FLG ink designated as “IA” contained 0.56 g/L FLG and 1.09 g/L of the stabilizer sodium deoxycholate (SDC), while the one designated as “IB” contained 0.58 g/L FLG, 1.13 g/L SDC, and traces (<0.003%) of N-methyl-2-2pyrrolidone (NMP) used in the graphite exfoliation process for the generation of FLG (Table 1). The inks were also provided in powdered form following further processing steps, designated as “IA+”, which contained 80% wt FLG and 20% wt SDC, and “IB+”, containing only FLG, with traces of DMSO and NMP (Table 1). To enable aquatic testing, stock suspensions of the FLG ink powders (IA+ and IB+) were prepared in sterile Milli-Q water in 30 mL Pyrex glass tubes (POBEL, Madrid, Spain), at concentrations of 1 g/L, and dispersed by sonication at 37 kHz for 30 min (total energy delivered: 36,826 J) in an ultrasonic cleaning bath (Fisherbrand S-series; Thermo Fisher Scientific, Waltham, MA, USA). To enable testing of the functional component (FLG) only, an additional processing step (washing) was performed on the powdered ink IA+, in an attempt to eliminate any additives, and this sample was designated “IA++”. Stabilizer vehicles were also supplied for testing and served as controls for the aqueous dispersions of FLG inks IA and IB (Table 1). These were designated as “VA”, which contained 1 g/L SDC, and “VB”, which contained 1 g/L SDC plus traces of NMP.

### 2.2. Characterization

#### 2.2.1. Dynamic Light Scattering

The hydrodynamic size distribution of the as-supplied aqueous dispersions of FLG inks (IA and IB) and ink powders in stock suspension (Milli-Q water) (IA+ and IB+) was determined by dynamic light scattering (DLS) analysis using a Zetasizer Nano-ZS apparatus (Malvern Instruments Ltd., Malvern, UK). The size distributions under test conditions (e.g., in aquarium water and cell culture medium) and over the specific test durations (e.g., over 96 h in aquarium water) were recorded to monitor their stability and/or any changes in properties (e.g., size, aggregation state, polydispersity index, zeta potential). At least four measurements (10 runs for each measurement, 10 s/run) were taken of each sample. The attenuator and the optimal measurement position were automatically determined in the first measurements of each concentration in each sample, and thereafter they were fixed and used to monitor stability (size distribution maintenance) [14]. Data were analyzed using Zetasizer Software version 6.34 (Malvern Instruments Ltd.). Z-potential measurements of some samples were also performed by electrophoretic light scattering using disposable capillary cuvettes (Malvern Instruments Ltd.). Three measurements were taken of each sample. The number of runs was set automatically by the device.

#### 2.2.2. Atomic Force Microscopy

To characterize the flake size of FLG during exposure of the inks to aquarium water, atomic force microscopy (AFM) was used. Samples (2 μL) of aqueous dispersions of FLG inks IA and IB diluted in aquarium water (6.34 mg/L) were taken at the start of the experiment (t0), transferred onto freshly exfoliated mica substrates, and air-dried at room temperature for 4 days. AFM imaging was performed in tapping mode on a Nanoscope V Multimode apparatus (Bruker, Billerica, MA, USA), using a TESP-SS tip with a radius of 2 nm and a spring constant of 42 N/m (Bruker). Height and phase images were recorded simultaneously. Images were analyzed using NanoScope software v1.40r1 (Bruker). Measurements of the lateral dimension of the FLG inks were performed on topographical AFM images.

#### 2.2.3. Turbiscan

Turbiscan (Formulaction, Scientific Instruments, Toulouse, France) enables the fast and sensitive identification of dispersions’ destabilization mechanisms (such as sedimentation, flocculation, and coalescence). A temperature-controlled measurement cell allows for stability monitoring under specific conditions. Stability is indicated by the slope of the variation in the transmission vs. time, with a higher slope indicating faster sedimentation. This technique provides additional information complementary to that obtained by other means, and it was used to monitor the stability of the FLG ink IA in aquarium water under different conditions, representative of waters without fish (clean water, CW), and of fish water at the start (fish water, FW0) and end of testing (fish water, FW96). Measurements were performed on ink IA because it represents the original unprocessed ink product and could give valuable information in this preliminary stability test. The FLG ink was also prepared at dilutions of 1/10 and 1/100 to assess if there was any influence of the material concentration on stability/destabilization in the system.

### 2.3. In Vitro Cytotoxicity Assays

To test the cytotoxicity of the aqueous dispersions of FLG inks (IA and IB), their vehicles (VA and VB), and the further processed and washed dispersed powders (IA+, IB+, and IA++), we used the cell line RTL-W1 derived from rainbow trout liver, which was kindly provided by Drs. Lee and Bols [15]. The toxicity was assessed using three assays based on different toxicological endpoints (AlamarBlue (resazurin) for metabolic activity, 5-carboxyfluorescein diacetate-acetoxymethyl ester (CFDA-AM) for cell membrane integrity, and neutral red dye uptake (NRU) for lysosomal function) [16]. This was the same set of biomarker assays used in the OECD TG 249, Fish Cell Line Acute Toxicity—the RTgill-W1 cell line assay [17]; however, instead of using the RTgill-W1 cell line and the L-15/ex medium, the RTL-W1 liver cell line was chosen for its capability to also monitor any effects on hepatic Cyp1a and Cyp3a enzyme activities (see Section 2.6 below), which play a key role in detoxification processes.

The cells were seeded (2.5 × 10^4^ cells/well) into transparent flat-bottomed 96-well plates (Greiner Bio-one GmbH, Frickenhausen, Germany) and incubated at 20 °C in Leibovitz´s L-15 medium (Gibco; Thermo Fisher Scientific) supplemented with 10% fetal bovine serum (FBS) (Sigma-Aldrich; Merck group, Darmstadt, Germany) and 1% (50,000 Units/500 mL) streptomycin/penicillin (P/S) (Lonza; Thermo Fisher Scientific). After 24 h post-seeding, the cells were exposed to aqueous dispersions (IA/IB/IA+/IA++/IB+) and vehicles (VA/VB) for 1 or 7 days. At least three independent experiments were performed for each test material and exposure time. Working concentrations of each material were prepared, either directly diluted from ink/vehicle dispersions or Milli-Q stock suspensions prepared from the ink powders, in complete L-15 medium (10% FBS, 1% P/S); 1:2 serial dilutions ranging from 0.4 to 100 mg/L were used to create a series of concentrations for testing. The samples were vortexed just before applying them to the cells. Vehicle controls and controls without any treatment were included. In addition, a positive control treated with a concentration range (15.6–500 μM) of sodium dodecyl sulfate (Sigma-Aldrich; Merck group) was also included in each plate. After the exposure period, the medium was removed, and the viability of the cells was evaluated according to a modified version [18] of a protocol described by Dayeh et al. (2005) [15]. Briefly, the cells were washed twice with PBS. The wells received 100 μL of 1.25% (*v*/*v*) AlamarBlue^TM^ (Invitrogen; Thermo Fisher Scientific) and 4 μM CFDA-AM (Invitrogen; Thermo Fisher Scientific) prepared in serum-free/phenol-red-free L-15 medium. The plates were incubated for 30 min at 20 °C in the dark, and fluorescence was measured on a Spark 20M microplate reader (Tecan, Männedorf, Switzerland) at a wavelength of 532/590 nm (excitation/emission) for AlamarBlue, and at 485/535 nm for CFDA-AM. The cells were washed with PBS and incubated with 100 μL of neutral red (NR) dye solution (33 μg/mL in serum-free/phenol-red-free L-15) for 1 h at 20 °C in the dark. After incubation, the cells were rinsed with PBS, and the retained dye was extracted with 100 μL of acidified (1% glacial acetic acid) 50% ethanol/49% Milli-Q water solution. Thereafter, fluorescence was measured at 532/680 nm. The fluorescence values were corrected by subtracting the blank measures and expressed as percentages of the control values. Potential interferences of the FLG dispersions with the fluorophores that are formed in the course of the cytotoxicity assays were assessed as previously described by Kalman et al. (2019) [19].

### 2.4. Measurement of Intracellular Reactive Oxygen Species (ROS)

Intracellular ROS levels were measured by the dichlorofluorescein (DCF) assay (using the 6-carboxy-2′7′dichlorodihydrofluorescein diacetate (DCFH-DA) probe) reported by Wang and Joseph (1999) [20]. Cell seeding and treatment with aqueous dispersions (IA/IB/IA+/IA++/IB+) and vehicles (VA/VB) were performed as described above. Cells treated with hydrogen peroxide (3.13–100 μM) were used as positive controls. After 1 or 7 days of exposure, the cells were washed twice with PBS and incubated with 100 μL of a 100 μM DCFH-DA probe (in serum-free/phenol-red-free L-15 medium) for 30 min in the dark at 20 °C. After the removal of DCFH-DA, the cells were washed twice with PBS and incubated with 100 μL of serum-free/phenol-red-free L-15 medium. Fluorescence was measured on a Spark 20M microplate reader (Tecan) at 485/530 nm (excitation/emission) immediately, and then every 10 min over 60 min. ROS levels were quantified on the basis of the percentage increase in fluorescence over time and are presented as percentages of the fluorescence of untreated control cells. Potential interferences of the FLG dispersions with the conversion product of the DCFH-DA probe (100 μM DCF) were tested in the same way as for the other assays [19].

### 2.5. Fish Acute Toxicity Test

Assessment of acute toxicity was performed according to OECD TG 203 [21]. Juvenile rainbow trout (*Oncorhynchus mykiss*) with an average weight of 1.3 ± 0.08 g and size of 5 ± 0.16 cm were obtained from a local trout farm (Felechosa, Asturias, Spain) and acclimated at the INIA-CSIC fish facilities (Carretera de la Coruña, km 7.5, Madrid, Spain). Prior to distribution into the experimental aquariums, the fish were kept for five days in a 450 L tank with a water recirculation system at 13.4 ± 0.4 °C, with a photoperiod of 12 h light/day. Once a day, the fish were fed with a commercial diet (BioMar Inicio Plus 801; BIOMAR, Aarhus, Denmark) for trout at a rate of 2% of their body weight. After five days, the fish were transferred to rectangular 33 L glass tanks (10 fish/tank) with filtered reconstituted water (according to OECD parameters) and allowed to acclimatize for 7 days under semi-static conditions prior to the start of the experiments. During this adaptation period, the fish were maintained under controlled conditions and fed with a commercial diet once a day. Feeding was stopped 24 h prior to the start of the experiments. Throughout the whole experiment, the test conditions and the water parameters were within the recommended OECD range, i.e., the photoperiod was 12/12 h, the temperature was 13.4 ± 0.4 °C, the conductivity was 236.7 ± 3.81 μS/cm, the water’s pH was 7 ± 0.1, and the dissolved oxygen remained above 60%. The survival at the end of the acclimatization period was 98.8%, and there was 100% survival for the control tanks throughout the whole experiment.

A preliminary test under static conditions was carried out by exposing the fish to the FLG dispersions (IA, IA+, IB, and IB+). In a second test round, we exposed the fish to the FLG inks (IA/IB) and the corresponding vehicles (VA/VB) for 96 h, while the reprocessed powders were not tested due to the low effects of the powders seen in the cytotoxicity assays. Only a maximum concentration (67.6 mg/L FLG) in a limit test was used for IA; however, a range of five concentrations (2.8, 6.3, 13.9, 30.7, and 67.6 mg/L FLG) with a 2.2 dilution factor was tested for IB, since this represents the ink formulation with all constituents present (i.e., FLG, SDC, and traces of NMP). Voyager Nano turbines (SICCE, Pozzoleone, Italy) were used in all of the aquariums to improve the stability and maintenance of the FLG ink dispersions in the water column. During the exposure period, the FLG concentrations were monitored by UV–vis spectrophotometry with a Spark 20M (Tecan) at 270 nm (peak wavelength) using the FLG inks’ (IA/IB) standard curves (0.8–430 mg/L). Absorbance at 270 nm was measured at the beginning of the experiment and every 24 h thereafter. Mortality, along with the fish’s appearance and swimming behavior, was checked at 0, 2, 5, and 24 h, and then twice per day until the end of the exposure period (96 h). At the end of the experiment, the fish were anesthetized with 100 mg/L of ethyl 3-aminobenzoate methanesulfonate (MS-222; Sigma-Aldrich), weighed, sized, and finally euthanatized by decapitation. Their gills and livers were dissected and stored −80 °C until the enzyme activity analyses.

### 2.6. EROD/BFCOD Activities

#### 2.6.1. Enzyme Activities in RTL-W1 Cell Cultures

Ethoxyresorufin-O-deethylase (EROD) and benzyloxy-4-trifluoromethylcoumarin-O-debenzyloxylase (BFCOD) activities were measured in RTL-W1 cells after 1 or 7 days of exposure, as previously described by Valdehita et al. (2023) [22] and Creusot et al. (2015) [23]. EROD and BFCOD activities are associated with Cyp1a and Cyp3a, which play essential roles in the oxidation of xenobiotics, and whose induction can be used as a biomarker of contaminant exposure. Cells were seeded in 96-well plates (Greiner Bio-one GmbH) at 2.5 × 10^4^ cells/well (EROD) or 5 × 10^4^ cells/well (BFCOD) and treated with aqueous inks (IA/IB), ink powder suspensions (IA+/IA++/IB+), and vehicles (VA/VB), as described above (Section 2.3). In this case, cells treated with benzo[k]fluoranthene (0.039 nM to 1 μM) were used as positive controls. After the exposure period, the cells were washed twice with PBS. Afterwards, 100 μL of 6 μM 7-ethoxyresorufin solution (EROD assay) or 80 μM 7-benzyloxy-4-trifluoromethyl coumarin (BFCOD assay) was added to each well, and fluorescence was measured immediately, and then every 10 min over 30–40 min, on a Spark 20M microplate reader (Tecan) at 532/590 nm (excitation/emission) for EROD activity and at 409/530 nm for BFCOD activity. The solution was removed and the cells were rinsed with PBS and frozen at −80 °C overnight. Thereafter, the total protein content in each well was determined by reconstitution of the frozen cell lysate in 75 μL of PBS, and then by adding 75 μL of 0.15 mg/mL fluorescamine solution in acetonitrile. The fluorescence was measured at 360/450 nm (excitation/emission) after 30 min of incubation in the dark. EROD activity was expressed as pmol of resorufin produced in 1 min per mg of protein, while BFCOD activity was expressed as pmol of 7-hydroxy-4-trifluoromethyl coumarin (HFC) produced in 1 min per mg of protein. The pmol concentrations of resorufin and HFC were calculated using the respective standard curves. Protein contents were calculated using a bovine serum albumin (BSA) standard curve.

#### 2.6.2. Enzyme Activities in Rainbow Trout Livers

Ethoxyresorufin-O-deethylase (EROD) and BFCOD activities were also monitored in the livers of rainbow trout through the in vivo acute toxicity test with aqueous FLG ink dispersions (IA/IB) and vehicles (VA/VB) (see Section 2.5). Tissue fragments were homogenized in 250 μL of ice-cold homogenization buffer (0.1 M Tris HCL pH 7.5, 1 mM EDTA, 0.25 M sucrose, 150 mM KCL, 20% *v*/*v* glycerol, 1 mM DTT, and 5 μg/mL of pepstatin A, aprotinin, and leupeptin) using a TissueLyser II (Qiagen, Venlo, the Netherlands) for 30 s at 30 Hz. The homogenates were then centrifuged at 6000× *g* for 10 min at 4 °C, and the supernatants were centrifuged at 16,000× *g* for 60 min at 4 °C. The resulting pellets were dissolved in 100 μL of homogenization buffer and used for EROD and BFCOD analyses.

EROD activity was measured at room temperature following the methodology established by Burke and Mayer (1974) [24], and as described by Habila et al. (2017) [25]; 96-well plates (Greiner Bio-one GmbH) were used for carrying out the measurements. Each sample (10 μL) was analyzed in duplicate. The reaction was followed by reading the fluorescence every 10 min over 30–40 min on a Spark 20M microplate reader (Tecan) at 532/590 nm (excitation/emission). A resorufin standard curve was used to quantify the pmol of resorufin formed during the assay. Sample protein concentrations were quantified using a fluorescamine-based assay [26] and a BSA standard curve. EROD activity was expressed as pmol resorufin/min/mg protein.

BFCOD activity was measured at 30 °C as described by Thibaut et al. (2006) [27] in 96-well plates (Greiner Bio-One GmbH). The reaction was monitored in duplicate (10 μL undiluted samples), reading the fluorescence every 10 min over 30–40 min on a Spark 20M microplate reader (Tecan) at 409/530 nm (excitation/emission). HFC pmol production was calculated using a standard curve. Protein concentration was quantified using a fluorescamine-based assay. BFCOD activity was expressed as pmol HFC/min/mg protein.

### 2.7. Statistical Analysis

Data are expressed as the mean ± standard error of the mean (SEM). All statistical analyses were performed using Prism 5.0 (GraphPad Software, Inc., La Jolla, CA, US). The normality of the distribution was confirmed by the Shapiro–Wilk test, and the homogeneity of variance was confirmed by Bartlett’s test. Significant differences between the control and treated groups were tested by one-way analysis of variance (ANOVA) (*p* < 0.05), followed by a post hoc Dunnett’s multiple comparisons test. When necessary, comparisons between two groups were performed using Student´s *t*-test.

## 3. Results

### 3.1. Characterization of FLG Inks

#### 3.1.1. Dynamic Light Scattering

The estimation of the hydrodynamic diameters (HDDs) and the average size (Z-ave) of the different FLG dispersions diluted in Milli-Q water, complete L-15 culture medium, or aquarium water was performed using DLS. The analysis showed HDD values in the same range for FLG IA and IB, regardless of the dispersion medium used (~500 nm) (Table 2). In Milli-Q water dispersions, the HDD of the FLG ink suspensions (IA/IB) was lower than for the dispersed powders (IA+/IB+). The same was observed for the Z-ave size, suggesting better dispersion and less aggregation of FLG in the inks provided already in dispersion and with higher concentrations of SDC. In complete L-15 medium, characteristic FLG HDD peaks of the same size were measured and, again, smaller Z-ave sizes were recorded for the IA and IB inks at t0 (Table 2). After 7 days in L-15 medium, a generalized decrease in Z-Ave and HDD was observed in all cases, probably due to the larger particles having already been precipitated.

In aquarium water, we observed a mild and gradual increase in the Z-ave value during the fish acute toxicity test (see below), suggesting the formation of aggregates/agglomerates as time passed in both inks (IA/IB). At the beginning of the experiment, the HDD was lower for IA FLG inks compared with IB FLG inks (265 vs. 507 nm), but as the time passed the HDD reached similar values in both inks after 96 h (~500 nm). This is the same HDD as measured in Milli-Q water, indicating that under experimental conditions the FLG material maintained its size, with no important transformations. The Z-potentials of both inks had the same values at the beginning of the exposure period (−28 mV), but they decreased slightly after 96 h (−21.3 and −25.6 mV, respectively), indicating a decrease in stability during the experiment (Table 2). The high polydispersity index observed in all of the samples analyzed was indicative that we had a high degree of heterogeneity in particle size in all FLG dispersions.

#### 3.1.2. Atomic Force Microscopy

**Figure 1:**
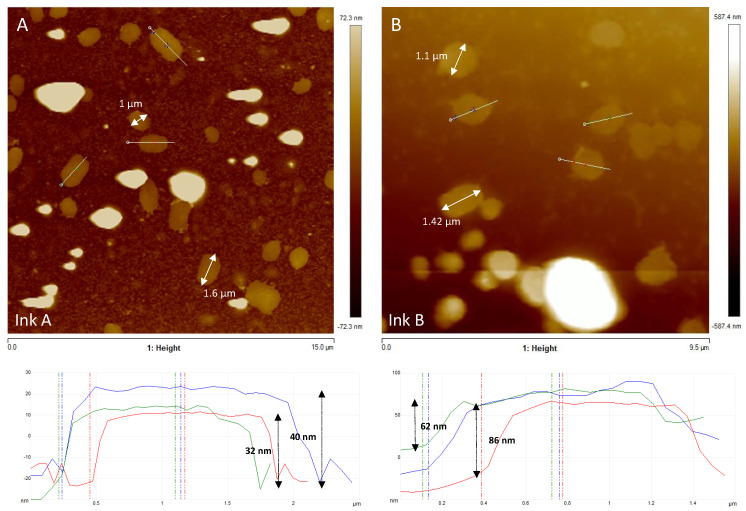
AFM topographic images of FLG ink suspensions. Both diluted FLG inks (IA and IB) (6.34 mg/L) contain graphene layers of heterogeneous size. The lateral dimensions of some graphene layers are displayed in the images (white arrows). Height measurements performed on FLG inks (**A**,**B**) showed that there were several layers (ink (**A**) ~36 nm; ink (**B**) ~74 nm). Examples of height profiles (blue, green and red lines) are presented below the corresponding topographic images (section along the white line visible in the AFM image).

AFM analysis of the FLG inks (IA/IB) showed that most of the FLG IB ink flakes had a thickness of ~74 nm (Figure 1B) and were approximately twice the height of the FLG IA flakes (~36 nm) (Figure 1A), indicating the existence of several layers. Taking into account that the theoretical distance between layers is 0.34 nm, it seems that particles of this size must correspond with agglomerates. Regarding the lateral dimensions of the FLGs, the analysis showed that there was a distribution of sizes ranging between 0.8 and 2 µm in both cases (Figure 1).

#### 3.1.3. Turbiscan

**Figure 2:**
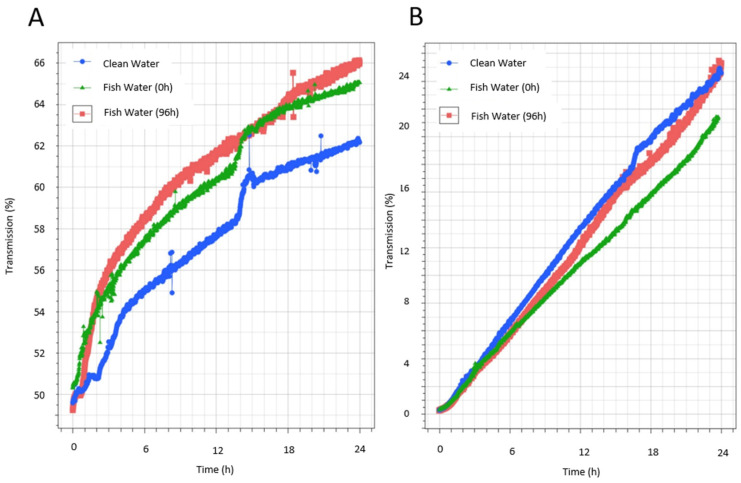
Turbiscan analysis of FLG inks’ destabilization kinetics in aquarium water. Measurement of FLG IA’s stability in different aquarium water, representing water at the start of the experiment without fish (clean water, CW), water that had fish during acclimatization (fish water, FW0), and water that had maintained fish for 96 h (fish water, FW96). Comparison of the variation in the mean transmission (whole height 4 mm–36 mm) at dilutions of 1/100 (**A**) or 1/10 (**B**) in CW, FW0, and FW96.

This analysis enables the sensitive identification of dispersions’ destabilization mechanisms, such as sedimentation. Stability is indicated by the slope of the variation in the transmission vs. time, with a higher slope indicating faster sedimentation. Results from the Turbiscan analysis of suspensions of the unprocessed ink product IA prepared in aquarium water showed that while the water condition influenced the stability, it also depended on the concentration of the ink. The influence of the water condition on stability was most obvious when ink IA was prepared at low concentrations (1/100 dilution). The kinetics of destabilization differed according to the water conditions (clean water, or fish water at t0 and t96), with a faster destabilization in waters that held fish during acclimatization (FW0) and for longer periods (matured water) (FW96) than in clean water (Figure 2A). However, this trend was not seen when FLG ink IA was prepared at high concentrations (1/10 dilution) (Figure 2B). In this case, the same kinetics was measured in the different waters over the first 6 h, after which some differences in destabilization emerged over time, especially in the case of FW0. In this case, the fastest destabilization was measured in clean water, while the slowest was measured in fish water during acclimatization (FW0). This highlights the complex roles of the different concentration ratios of substances released by the fish, the concentrations of FLG, and dynamic interactions over time that must be monitored in the test system.

### 3.2. In Vitro Cytotoxicity in RTL-W1 Cells

The cytotoxicity of the FLG dispersions was assessed in a rainbow trout cell line of hepatic origin of (RTL-W1). Before performing the cytotoxicity assays, potential interferences of the FLG dispersions and vehicles with the cytotoxicity assays’ reagents were tested in the presence of cells. No interference of the FLG dispersions with the AlamarBlue, CFDA-AM, NRU, or ROS assay reagents in L-15 medium was detected.

#### 3.2.1. AlamarBlue Assay

**Figure 3:**
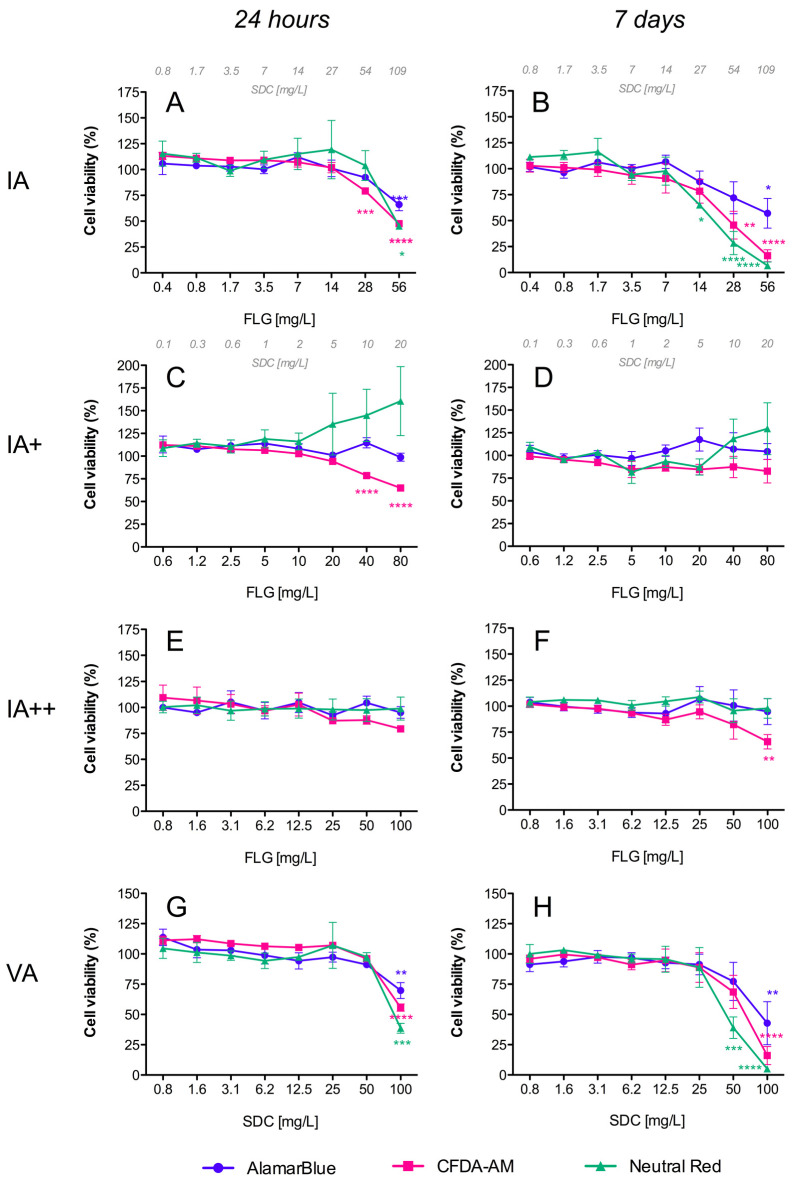
Cytotoxicity of FLG A’s dispersions in RTL-W1 cells. Effects of increasing concentrations of FLG IA (**A**,**B**), IA+ (**C**,**D**), IA++ (**E**,**F**), and VA (**G**,**H**) after 24 h and 7 days. Dispersions were prepared by diluting aqueous stocks in complete L-15 medium. Cells incubated with only medium served as negative controls (100%). Effects of the treatments were expressed as percentages of the control values. Cytotoxicity was evaluated by the AlamarBlue assay, CFDA-AM assay, and NRU assay. An extra *x*-axis (top) has been plotted in (**A**–**D**) to indicate the SDC concentrations present in the dispersions. Lines and symbols represent the mean ± SEM of at least three independent experiments. Statistically significant differences with respect to the control group (one-way ANOVA, Dunnett’s multiple comparisons test) are indicated as follows: * *p* < 0.05, ** *p* < 0.01, *** *p* < 0.001, **** *p* < 0.0001.

**Figure 4:**
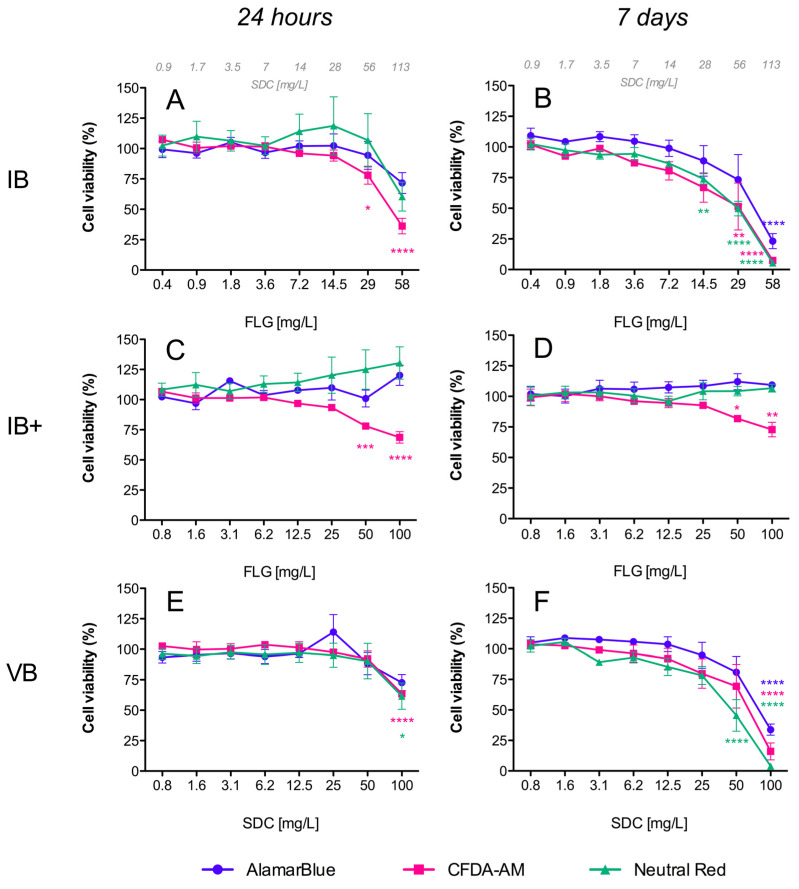
Cytotoxicity of FLG B’s dispersions in RTL-W1 cells. Effects of increasing concentrations of FLG IB (**A**,**B**), IB+ (**C**,**D**), and VB (**E**,**F**) after 24 h and 7 days. Dispersions were prepared by diluting aqueous stocks in complete L-15 medium. Cells incubated with only medium served as negative controls (100%). Effects of the treatments were expressed as percentages of the control values. Cytotoxicity was evaluated by the AlamarBlue assay, CFDA-AM assay, and NRU assay. An extra *x*-axis (top) has been plotted in (**A**,**B**) to indicate the SDC concentrations present in the ink dispersions. Lines and symbols represent the mean ± SEM of at least three independent experiments. Statistically significant differences with respect to the control group (one-way ANOVA, Dunnett’s multiple comparisons test) are indicated as follows: * *p* < 0.05, ** *p* < 0.01, *** *p* < 0.001, **** *p* < 0.0001.

Resazurin (7-Hydroxy-3H-phenoxazin-3-one 10-oxide), the active compound in the commercial solution AlamarBlue^TM^, is a redox indicator that yields a fluorescent signal in response to metabolic activity. It is reduced to the fluorescent compound resorufin, and a decrease in fluorescence is indicative of diminished metabolic activity and cytotoxicity. In our study, exposure of RTL-W1 cells to FLG inks (IA/IB) and their respective vehicles (VA/VB) resulted in a significant dose- and time-dependent decrease in fluorescence intensity (Figure 3A,B and Figure 4A,B), but not for the reprocessed ink powders (IA+/IA++/IB+) (Figure 3C–F and Figure 4C,D). The decreases in metabolic activity were very similar for the inks (IA/IB) and their respective vehicles (VA/VB) (Figure 3A,B vs. Figure 3G,H and Figure 4A,B vs. Figure 4E,F), being significantly different from the negative control at the higher doses used and more pronounced at 7 days (cell viability IA: 57%, VA: 42%, IB: 23%, VB: 34%) (Figure 3B–H and Figure 4B–F). At this point, it is important to highlight that the concentration of SDC in both inks and vehicles was in the same range (Figure 3B,H and Figure 4B,F). Although not significant, the data after 7 days suggest a greater reduction in metabolic activity for IB (the ink with NMP traces) than for IA (cell viability: IB 23% vs. IA 57%) (Figure 3B and Figure 4B).

#### 3.2.2. CFDA-AM Assay

This assay is based on the conversion of CFDA-AM to its fluorescent product 5-carboxyfluorescein (5-CF) by cytosolic esterases, which are only retained in cells with an intact plasma membrane. In our study, a dose- and time-dependent decrease in fluorescence was detected for the inks (IA/IB), reprocessed ink powders (IA+/IA++/IB+), and vehicles (VA/VB), which was indicative of membrane disruptions in all cases (Figure 3 and Figure 4). The cytotoxic effects were higher for the inks (IA/IB) (Figure 3A,B and Figure 4A,B) and vehicles (VA/VB) (Figure 3G,H and Figure 4E,F) than for the powders (IA+/IA++/IB+) (Figure 3D–F and Figure 4D), and this was most evident after 7 days of exposure. Although not significant, the effect with the higher doses after 7 days was slightly higher for IB than for IA (cell viability: IB 7% vs. IA 16%) (Figure 4B and Figure 3B) and for IB than for VB (cell viability: IB 7% vs. VB 16%) (Figure 4B,F). Also, interestingly, there was a significant loss in cell viability according to the plasma membrane disruption at concentrations ≥ 40 mg/L FLG for the reprocessed inks IA+ and IB+ after 24 h (Figure 3C and Figure 4C, respectively).

#### 3.2.3. Neutral Red Uptake (NRU) Assay

NR is a fluorescent probe that accumulates in the lysosomes of viable cells. In cells with damaged lysosomal function, less NR is taken up and/or retained. In our study, a dose- and time-dependent decrease in fluorescence was detected only for both inks (IA/IB) (Figure 3A,B and Figure 4A,B) and their respective vehicles (VA/VB) (Figure 3G,H and Figure 4E,F) after 24 h and 7 days of exposure, whereas no significant effect was observed for the reprocessed ink powders (IA+/IA++/IB+) (Figure 3C–F and Figure 4C,D). No significant differences in cell viability were detected between IA-VA and IB-VB (Figure 3B–H and Figure 4B–F), or between IA and IB with the highest doses used (Figure 3B and Figure 4B).

#### 3.2.4. Generation of Intracellular ROS

**Figure 5:**
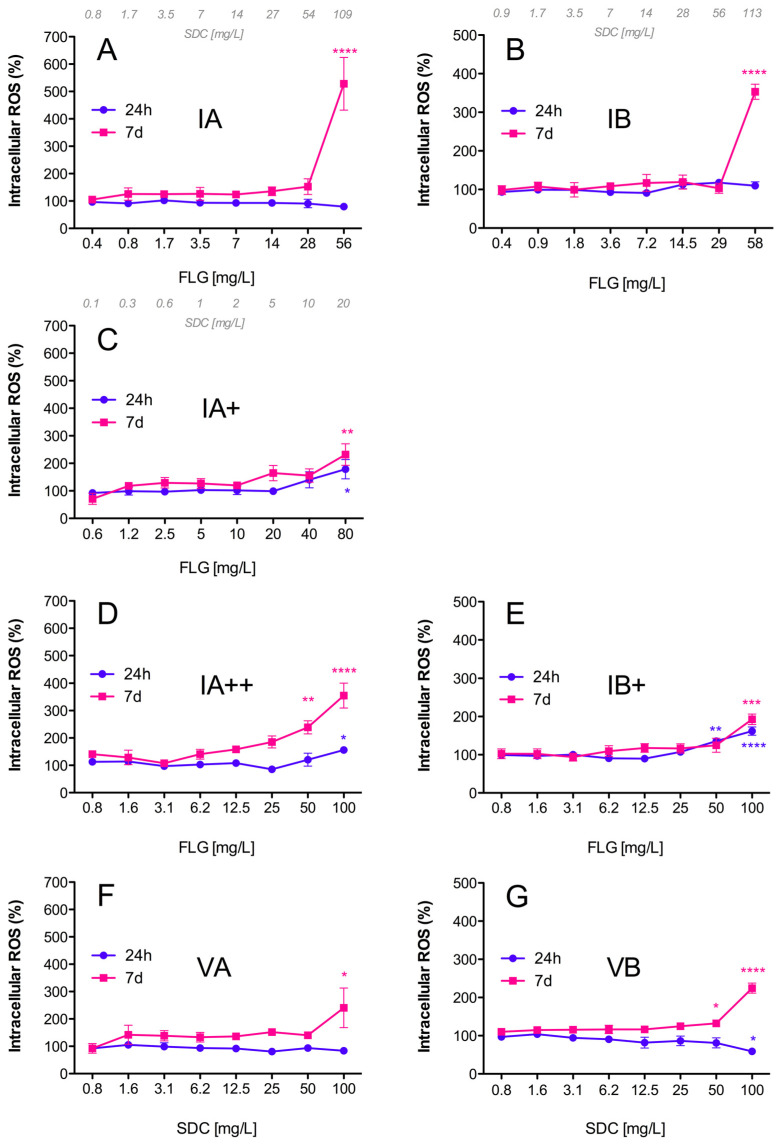
Intracellular reactive oxygen species (ROS) in RTL-W1 cells. Effects of increasing concentrations of FLG IA (**A**), IA+ (**C**), IA++ (**D**), VA (**F**), IB (**B**), IB+ (**E**), and VB (**G**) after 24 h and 7 days of exposure. Dispersions were prepared by diluting aqueous stocks in complete L-15 medium. Cells incubated with only medium served as negative controls (100%). The effects of the treatments are expressed as percentages of the control values. An extra *x*-axis (top) has been plotted in (**A**–**C**) to indicate the SDC concentrations present in the dispersions. Lines and symbols represent the mean ± SEM of at least three independent experiments. Statistically significant differences with respect to the control group (one-way ANOVA, Dunnett’s multiple comparisons test) are indicated as follows: * *p* < 0.05, ** *p* < 0.01, *** *p* < 0.001, **** *p* < 0.0001.

Inks (IA/IB), reprocessed ink powders (IA+/IA++/IB+), and vehicles (VA/VB) induced intracellular ROS formation in RTL-W1 cells in a dose- and time-dependent manner (Figure 5). After 24 h of exposure, statistically significant increases in fluorescence were only observed for reprocessed powders IA+, IA++, and IB+ (Figure 5C–E). At the highest exposure doses, the IA+, IA++, and IB+ powders increased the intracellular ROS levels in the cells to 178, 156, and 161% with respect to the control, respectively (Figure 5C–E). These levels were maintained following 7 days of exposure to IA+ and IB+ (Figure 5C,E), and they increased to 355% in the case of the IA++ powder, which was reprocessed and washed to ensure that it contained only FLG (Figure 5D). On the other hand, the exposure to IA and IB did not show any effects after 24 h, but the intracellular ROS formation increased drastically after 7 days, reaching levels of 528% (IA) and 353% (IB) with respect to the controls at the highest exposure doses (Figure 5A,B). The vehicles VA and VB also induced ROS generation at high exposure doses after 7 days, reaching 240% and 224% increased ROS levels with respect to the control, respectively (Figure 5F,G). The levels of ROS formation were higher for the inks, which contained FLG and SDC (IA/IB), than for the vehicles containing only the SDC and NMP solvents (VA/VB).

#### 3.2.5. EROD/BFCOD Activities

None of the FLG dispersions or vehicles induced EROD and BFCOD activities in RTL-W1 cells during the exposure period. The basal EROD and BFCOD activities in the control cells ranged between 3.43 ± 0.51 and 0.75 ± 0.07 pmol/min/mg protein, respectively, and these values were similar in the treated cells.

### 3.3. In Vivo Toxicity Tests in Rainbow Trout

#### 3.3.1. Fish Acute Toxicity Test

The acute toxicity test was developed following the requirements described in OECD TG 203. In a preliminary test, we detected that all of the FLG dispersions (IA, IA+, IB, and IB+) were highly unstable in aquarium water, precipitating an important quantity of the FLG (>25% in all cases) after 24 h. Even with this loss in concentration, the FLG ink dispersion IB caused 28.5% mortality at the highest nominal concentration, tested in a limit assay with only this concentration (58 mg/L FLG, 113 mg/L SDC and NMP traces). A similar mortality level (28.5%) was observed for IB+ (100 mg/L FLG, traces of DMSO and NMP). We decided to repeat the in vivo test under static conditions, using an entire range of concentrations according to TG 203’s indications. Additionally, turbines were applied to the aquariums in order to generate a stream of water that would improve the FLG dispersions’ maintenance in the water column (stability). Tests were performed with both aqueous dispersed inks (IA/IB) and their vehicles (VA/VB), while the reprocessed powders were not tested due to the low effects of the powders seen in the cytotoxic assays. We tested high concentrations of IA (67.6 mg/L FLG, 131.5 mg/L SDC) and the vehicles VA and VB (100 mg/L each), along with a complete series of five concentrations of IB (2.88, 6.34, 13.96, 30.7, and 67.6 mg/L FLG), the ink for which showed some mortality in the preliminary test, in order to calculate a potential lethal concentration (LC).

**Figure 6:**
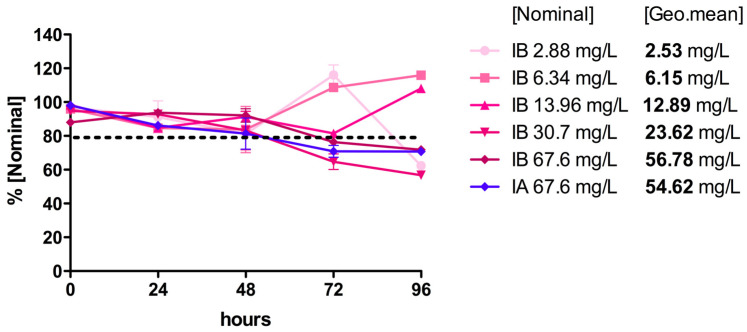
Concentrations of FLG inks IA and IB throughout the acute toxicity test. The FLG ink concentrations in each aquarium were determined by UV–vis spectrophotometry (270 nm) every 24 h. Values of FLG inks IA and IB are expressed as percentages of the nominal concentrations. To determine the real FLG concentrations to which the fish were exposed during the test, a geometric average was also calculated.

The FLG concentrations in each aquarium were monitored by UV–vis spectrophotometry at the beginning of the experiment, and then every 24 h thereafter (Figure 6). After 24–48 h, a gradual precipitation of the FLG content was observed in all cases. Following the recommendation (TG 203) to keep the FLG concentrations in the water column between 80 and 120% of the nominal concentration, and assuming that the observed loss of concentration was due to sedimentation, small volumes of concentrated ink dispersions were added during the first 72 h to re-establish the concentrations when necessary. All of the doses were maintained within this range except during the last 24 h, when the doses of 67.6, 30.7, and 2.88 mg/L FLG dropped to 70%, 57%, and 62% of the nominal concentration, respectively (Figure 6). To determine the real FLG concentrations to which the fish were exposed in each aquarium, and following the indications of OECD GD 317 [28], a geometric mean of the measured concentrations was calculated (Figure 6).

After 96 h, only one fish had died (10% mortality) under exposure to IB at a nominal concentration of 30.7 mg/L FLG/60 mg/L SDC (23.62 mg/L FLG, geo. mean); however, no mortality was detected at higher nominal concentrations of IB or at the tested concentrations of IA (67.6 mg/L FLG nominal, 56.78/54.62 mg/L FLG, geo. mean), indicating a lack of dose-dependent mortality. No visible abnormalities with regards to equilibrium and swimming behavior were detected in the control and treated groups at nominal doses ≤ 13.96 mg/L. Above 30.71 mg/L FLG, it was difficult to properly observe the fish in the aquariums, due to the darkness of the water with this test material. No changes in the pH, dissolved oxygen, or temperature were observed during the test.

#### 3.3.2. EROD/BFCOD Activities in Livers and Gills

**Figure 7:**
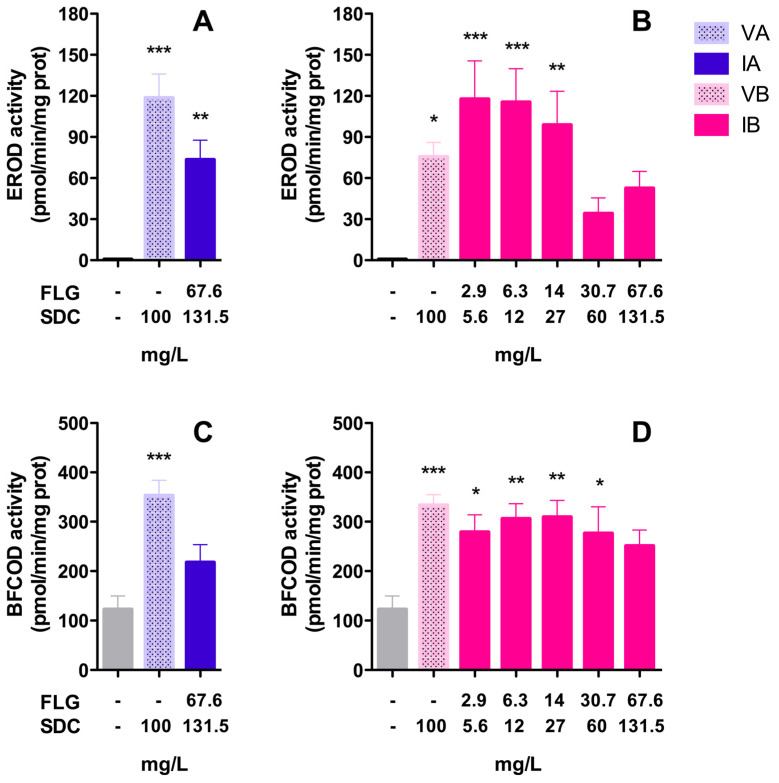
Enzyme activities in rainbow trout livers: EROD (**A**,**B**) and BFCOD (**C**,**D**) activities after 96 h of exposure to FLG inks. Bars represent the mean (n = 7) values ± SEM of the following groups: control, VA, IA, VB, and IB. Statistically significant differences with respect to the control group (one-way ANOVA, Dunnett’s multiple comparisons test) are indicated as follows: * *p* < 0.05, ** *p* < 0.01, *** *p* < 0.001.

Significant increases in EROD and BFCOD activities with respect to the control group were detected in the livers of fish after 96 h of exposure (Figure 7). The analyses of EROD activities showed that both vehicles (VA/VB) and inks (IA/IB) were able to stimulate Cyp1a enzyme activity. VA (100 mg/L SDC) provoked an increase of more than 180-fold compared to the control group’s activity, reaching 118 pmol resorufin/min/mg protein, while with the ink IA (nominal concentration: 67 mg/L FLG–131.5 mg/L SDC) the activity reached 73 pmol resorufin/min/mg protein (Figure 7A). VB (100 mg/L SDC) and the lower doses of the ink IB (nominal concentrations: 2.9, 6.3, and 14 mg/L FLG) provoked significant increases in EROD activity (Figure 7B). The maximum increase in enzyme activity was detected with the lower dose of ink B (nominal concentration: 2.9 mg/L FLG–5.6 mg/L SDC), reaching 117 pmol resorufin/min/mg, which decreased as the concentration of IB increased, until reaching a minimum with the doses of 30.7 and 67.6 mg/L of FLG (Figure 7B). Regarding BFCOD activity, the analysis showed that both vehicles (VA/VB) and only ink IB were able to significantly increase (2–3-fold) the production of HFC with respect to the control group (Figure 7C,D). The vehicle VA (100 mg/L SDC) promoted higher BFCOD activity than that seen with the ink IA (nominal concentration: 67 mg/L FLG–131.5 mg/L SDC). VB and the serial dilutions of IB significantly promoted the BFCOD activity in the same range, but no dose-dependent increases were detected (Figure 7D). No significant increases in EROD and BFCOD activity could be detected in the gills, and the values obtained in the treated groups were similar to those for the control group. The basal EROD and BFCOD activities in the gills control ranged between 1.24 ± 0.19 and 9.9 ± 0.8 pmol/min/mg protein, respectively.

## 4. Discussion

In this study, we assessed the hazard of a commercial FLG ink product using a fish acute toxicity test (OECD TG 203) and an in vitro model test system (fish cell lines). Using an SSbD approach, the product was supplied for testing in diverse formulations following different and additional processing steps. In total, five formulations were tested, and they consisted of two aqueous dispersed inks produced by different methodologies with the stabilizer SDC and solvent NMP (IA and IB), the same inks reprocessed, dried, and provided in powder form but which were later suspended for testing (IA+ and IB+), and the respective vehicles used in the preparation of IA and IB (VA and VB). A washed FLG powder A (IA++) was also prepared as a pure FLG ink product to distinguish between the effects of FLG and solvents (e.g., SDC and NMP/DMSO).

In the first place, we characterized the different FLG dispersions prepared as stocks in Milli-Q water and under test conditions via DLS, AFM, and Turbiscan. Although DLS is not ideal for 2D nanomaterials, it can provide information about changes in the aggregation/agglomeration state of the particles during the experiment [29]. The DLS results confirmed that the FLG dispersions with greater contents of the stabilizer SDC had lower HDD (IA/IB < IA+ < IA++/IB+), suggesting higher stability and less aggregation. Interesting results on the influence of the water condition on stability were provided by performing Turbiscan analysis of the ink IA. The findings from this analysis allowed us to conclude that the presence of fish (and their secretions, etc.) in test water and over the test duration (96 h) led to faster destabilizations when testing suspensions at low concentrations (dil. 1/100), while this had less effect when testing higher concentrations (dil. 1/10). Thus, both the test material concentration and the water condition dictate the extent of stability in the system, and this must be monitored. Material stability is a very important parameter, and concentration maintenance (≥80%) during testing must be measured and achieved as much as possible, to meet TG 203’s standardized test validity criteria. In this study, while maintenance above 80% was not achieved throughout the entire test, the exposure concentration was measured at various time intervals, which allowed a geometric mean approach to be used (OECD GD 317). In our system, after 96 h, the highest dose (67.6 mg/L FLG) dropped to 70% of the nominal test concentration, but using our approach a geometric mean concentration of 56.78 and 54.62 mg/L of FLG IA and IB, respectively, could be calculated to determine the real exposure.

When performing the hazard assessment of the studied materials, first, in an alternative approach to animal use, and in keeping with the 3 Rs principle of Replacement, Reduction, and Refinement in animal testing, we assessed their in vitro cytotoxicity using the cell line RTL-W1, which is derived from the epithelial cells of rainbow trout livers. This approach generated important information and permitted us to reduce the number of animals used, supporting the 3 Rs concept. In fact, the use of a rainbow trout cell line (RTgill-W1) in a fish acute toxicity test has recently been standardized and detailed in an OECD test guideline (TG No. 249) for use in aquatic toxicity assessment [17]. Instead of using this gill cell line, we chose a rainbow trout cell line of hepatic origin, since the liver plays an essential role in detoxification processes and there has been evidence to suggest potential interplay between graphene, AhR, and cytochromes with high activity at the hepatic level [22,30]. We used a combination of three cellular viability assays based on different toxicity endpoints (AlamarBlue, NRU, and CFDA-AM), as described in OECD TG 249, but we also included an intracellular ROS assay. We evaluated the effects following short-term (24 h) and longer-term (7 days) exposure to all of the FLG ink dispersions and the reprocessed ink powders and vehicles, and the results showed that both inks (IA/IB) and the respective vehicles (VA/VB) are potentially more toxic than the powders. The FLG inks (IA/IB) and their vehicles (VA/VB), but not the reprocessed ink powders (IA+/IA++/IB+), provoked a decrease in metabolic activity (AlamarBlue assay) in a dose- and time-dependent manner. In the same way, both inks (IA/IB) and vehicles (VA/VB) (but not the reprocessed ink powders) caused an increase in damage to lysosomal function (NRU assay) in a dose- and time-dependent manner, since less NR was taken up and/or retained by the lysosomes after exposure. However, in the CFDA-AM assay, the reprocessed ink powders (IA+/IA++/IB+), inks (IA/IB), and vehicles (VA/VB) all decreased fluorescence in a dose- and time-dependent manner, indicating that all of them provoked plasma membrane disruptions. This occurred with different intensities, and the effect was much higher for the inks (IA/IB) and vehicles (VA/VB) compared to the reprocessed ink powders (IA+/IA++/IB+). Plasma membrane damage can be a consequence of various cytotoxic effects. In the case of inks and vehicles, the levels of cytotoxic effect measured using this CFDA-AM assay corresponded to those evidenced in the AlamarBlue and NRU assays measuring metabolic disruption and lysosomal damage. However, for the reprocessed powders, cytotoxic effects were only measured using this assay, suggesting a particular effect of FLG on cellular membranes. One must also consider the potential differences in dosimetry within this submerged cell culture system between the ink suspensions and dispersed powders with different stabilities. In our study, we used a light microscope to observe the formation of a deposition of FLG on the cell surface, forming a layer that partially covered the cells’ surface when we used a high concentration (50–100 mg/L FLG). This could have caused a loss of structural integrity due to a strong physical interaction of FLG with the plasma membrane. Moreover, the presence of the ionic detergent SDC in IA+ (while much less than in IA/IB) and traces of DMSO/NMP in IB+ could also potentiate effects on the membrane integrity. However, one must also bear in mind that plasma membrane damage caused by graphene nanomaterials has been reported previously in both prokaryotic [31,32] and eukaryotic cells [18,19,22], and these materials’ physical interaction with cell membranes has been suggested as being one of the major causes of graphene cytotoxicity [33]. In general, the values obtained in our in vitro study showed that the inks (IA/IB) and vehicles (VA/VB) were more cytotoxic than the reprocessed ink powders (IA+/IA++/IB+), and only in terms of the plasma membrane integrity (CFDA-AM assay) were the inks (IA/IB) slightly more toxic than the vehicles (VA/VB), which would imply that one of the main contributors to the evidenced cytotoxicity was the stabilizer SDC.

In addition, the induction of oxidative stress is considered to be another of the principal mechanisms underlying nanomaterials’ toxicity [34,35]. In our study, all of the ink formulations tested (IA/IA+/IA++/VA/IB/IB+/VB) induced an increase in intracellular ROS levels. During the first 24 h, the effect was very modest and limited only to the FLG powders (IA+/IA++/IB+), but after 7 days of exposure all of the formulations (including the vehicle controls) increased the ROS levels, reaching the highest values with the inks (IA/IB). In other studies, with different cell lines, GO was also unable to induce the formation of intracellular ROS for periods of less than 24 h [18,36]. However, longer exposures to GO produced a strong induction of ROS in RTL-W1 cells after 7 days [22]. The particular mechanism through which a nanomaterial exerts oxidative stress is difficult to identify, but one of the most important nanomaterial-triggered mechanisms leading to increased intracellular ROS formation is probably the impairment of mitochondrial function [18]. Damage to mitochondria can directly affect the metabolic activity of cells, and in this study we observed that the FLG ink formulations (IA/IB) and vehicles (VA/VB) produced the same extent of decrease in metabolic activity in RTL-W1 cells; however, the induction of intracellular ROS was higher in cells exposed to the inks (IA/IB). All of these results support the idea that both ink formulations (IA/IB) were more cytotoxic than the respective reprocessed powders (IA+/IA++/IB+) and slightly more cytotoxic than the vehicles (VA/VB). The reason for these results could lie in toxic effects due to the solvents in the vehicles and the sum of the effects of FLG and the SDC in the ink formulations (IA/IB), with a much lower hazard profile associated with the much lower levels of solvents in the reprocessed ink powders.

To confirm the results observed using the in vitro fish cell line approach for acute toxicity assessment, we performed an in vivo acute toxicity test to assess the hazard of the FLG ink formulations (IA/IB). Due to the low effects of the powders seen in the cytotoxicity assays, we decided to test only the FLG inks (IA/IB) and their vehicles (VA/VB). We used juvenile rainbow trout, following the OECD TG 203 guidelines. In a preliminary limit test, we realized that a large portion of the FLG material (>25%) precipitated out of the water column after 24 h. According to TG 203, the concentration of the chemical being tested should be maintained at ≥80% of the nominal concentration throughout the test. For this reason, we decided to introduce turbines in all of the aquariums to improve the stability of the FLG dispersions, and the FLG concentrations in the water column were monitored every 24 h. The turbines were able to slightly increase the stability of FLG, although not enough to keep the concentrations above 80% throughout the entire test. Nevertheless, a geometric mean was calculated to represent the real exposure concentrations (OECD GD 317) in all of the aquariums, according to which the highest concentrations tested were 54.62 and 56.78 mg/L for IA and IB, respectively. Only one fish died after 96 h of exposure to 30.7 mg/L FLG (23.62 mg/L geometric mean) for IB, but no mortality was detected at any other concentration, and no dose-dependent effect was observed. Although the cytotoxicity detected in vitro was not reflected in the in vivo toxicity, the results obtained in vitro gave us valuable information that served to identify processes within an SSbD approach with increased hazard potential. While the levels of stabilizers in the exposure dose in vivo did not lead to mortality, sublethal effects were evidenced. Thus, the in vitro cell line served as an appropriate tool for an assessment of the environmental hazard (particularly to detect cytotoxic effects in fish at the cellular level) associated with specific ink formulations. This could translate to detrimental effects with increased exposure levels in fish, or if certain organ systems are exposed (e.g., the liver). The in vitro cell line approaches are an excellent alternative, and if they cannot be directly used for regulatory purposes they could, at least, serve for the assessment in a weight-of-evidence approach avoiding the use of living organisms according to the 3 Rs principle, as well as in the speeding up of product evaluation processes. In fact, within an SSbD framework, the use of the in vitro fish cell line NAM is most useful, even in the very early ideation phase of product conceptualization and innovation, providing a robust testing platform to enable early environmental hazards to be identified and prompt the need for rethinking. Through the presentation of this case study, a very good example has been shown of how even the decision to use stabilizers in a product’s formulation could greatly increase the product’s hazard profile. Thus, using test platforms such as the in vitro cell line test—both as an early warning for hazard assessment, and to explore possible alternatives in an SSbD approach—becomes very attractive.

Although no mortality was detected in the in vivo test, the analyses of sublethal effects revealed an intense detoxification activity in the livers of animals exposed to inks (IA/IB) or vehicles (VA/VB), with a strong induction of EROD and BFCOD activities in the liver. In fish, EROD activity is associated with the enzyme Cyp1a, which plays an essential role in the oxidation of xenobiotics, and whose induction can be used as a biomarker of contaminant exposure. The expression of Cyp1a is regulated by the aryl hydrocarbon receptor (Ahr), and Cyp1a activation can be evidenced at the enzymatic level through the associated EROD activity [37]. In our study, both inks (IA/IB) and vehicles (VA/VB) were able to induce EROD activity by more than 100-fold with respect to the control group. The results revealed that the SDC present in the vehicles contributed substantially to this Cyp1a induction during in vivo exposure. Maximal EROD levels were observed after exposure to IB and VB. A dose-dependent decrease in EROD activity was observed in animals exposed to IB (Figure 7B). This behavior could suggest, on the one hand, an additive effect of SDC and FLG and, on the other hand, an inactivation of Cyp1a activity by saturation as the concentration of IB increased. It is also known that the cytochrome P450 superfamily can bio-activate certain xenobiotics to form reactive species that can react with moieties in the active site, leading to inactivation of its own cytochrome P450s [38].

In addition to EROD activity, BFCOD activity has also been commonly used to monitor Cyp3a induction in fish [25]. Cyp3a is considered to be another of the enzymes playing a crucial role in the hepatic metabolism of xenobiotics. The basal physiological values of BFCOD activities reported in other fish species range from 2 to 57.5 pmol/min/mg [25]. In our study, the basal levels in the control group were around 122 pmol/min/mg, and the inks (IA/IB) and vehicles (VA/VB) induced BFCOD activity (~3-fold), but with less intensity than the EROD activity. The maximum values measured were obtained with the vehicles VA and VB (353 and 333 pmol/min/mg, respectively), while all of the concentrations of IB had a similar induction, ranging from 251 to 309 pmol/min/mg. In this sense, there is evidence that points to the conclusion that BFC is not a specific substrate of Cyp3a, and that it could be metabolized by a variety of cytochrome P450 family members, including Cyp1a [23]. In any case, the obtained results corroborate the important contribution of solvents present in VA and VB to the observed toxicity.

## 5. Conclusions

The present study highlights that FLG ink dispersions tested at concentrations of ≤67.6 mg/L FLG (≤56.7 mg/L geometric mean) did not provoke mortality in juvenile rainbow trout, but they did lead to disturbances at the enzymatic level (i.e., strong induction of EROD and BFCOD activities) in liver tissues, along with cytotoxic effects (metabolic activity, plasma membrane disruption, and lysosomal function) in the rainbow trout liver cell line RTL-W1. These effects were higher in the inks (IA/IB) and the respective vehicles (VA/VB), indicating that these effects were probably driven and could be enhanced by the concentration of SDC used as a stabilizer. When the concentration of SDC in the inks was reduced or completely removed through reprocessing of the inks (IA+, IB+, and IA++), a loss of metabolic activity or lysosomal damage at concentrations as high as 100 mg/L was no longer observed. Although the FLG powders (IA+/IA++/IB+) were not completely exempt from inducing a certain degree of effects on cells (e.g., plasma membrane damage/ROS induction), they had a lower hazard profile. Therefore, all of the obtained results show that, through a reprocessing approach, graphene-based products could reduce their (environmental) toxicity, representing a paradigmatic case study for how a safe(r) and sustainable by design framework can be used in future nanomaterial/2D material product development. The in vitro approaches are an excellent alternative in the environmental hazard assessment. They can serve for assessment in a weight-of-evidence approach, avoiding the use of living organisms according to the 3 Rs principle, and of course as a very attractive tool for early warning or alternative exploration in the ideation phase of product development.

## Figures and Tables

**Table 1:** Description of the tested FLG product formulations and corresponding vehicle solvent controls.

Product	Supplied Form	Processing	Composition
IA	Aqueous dispersion	SDC exfoliation	Water, FLG, SDC
IA+	Powder	SDC exfoliation, evaporation, freeze-drying	FLG, SDC
IA++	Powder	SDC exfoliation, evaporation, freeze-drying, washing, filtration, freeze-drying	FLG
VA	Aqueous solution	Aqueous dispersion	Water, SDC
IB	Aqueous dispersion	NMP exfoliation, evaporation	Water, FLG, SDC, traces of NMP
IB+	Powder	Exfoliation, evaporation, DMSO treatment, freeze-drying	FLG, traces of DMSO and NMP
VB	Aqueous solution	Aqueous dispersion	Water, SDC, traces of NMP

Notes: + denotes an additional processing step in the production process, ++ denotes an additional processing step and also an additional washing step.

**Table 2:** Hydrodynamic size distribution of FLG dispersions.

Sample	Dispersion Medium	Time (h)	Concentration (mg/L)	Z-Ave ^a^ (nm)	PDI ^b^	Average HDD ^c^ nm ± SEM (%) ^d^		Z-Potential (mV)
						Peak 1	Peak 2	
IA	Milli-Q H_2_O	0	560	835 ± 127	0.86 ± 0.07	448 ± 38 (88)	133 ± 12 (12)	-
IA+	Milli-Q H_2_O	0	800	2138 ± 187	0.85 ± 0.09	770 ± 118 (100)	-	-
IA++	Milli-Q H_2_O	0	1000	2643 ± 395	0.88 ± 0.10	890 ± 147 (100)	-	-
IB	Milli-Q H_2_O	0	580	557± 16	0.55 ± 0.03	518 ± 39 (86)	188 ± 26 (14)	-
IB+	Milli-Q H_2_O	0	1000	5101 ± 480	0.75 ± 0.07	933 ± 140 (100)	-	-
IA	L-15	0	56	2647 ± 480	1.00 ± 0.00	553 ± 132 (93)	111 ± 33 (7)	-
IA	L-15	144	56	1681 ± 81	1.00 ± 0.00	196 ± 36 (95.30)	8.5 ± 3.1 (4.7)	-
IA+	L-15	0	80	5406 ± 15	0.94 ± 0.06	500 ± 90 (100)	-	-
IA+	L-15	144	80	1953 ± 198	0.93 ± 0.07	388 ± 92.2 (100)	-	-
IA++	L-15	0	100	3032 ± 260	0.80 ± 0.14	606 ± 30 (100)	-	-
IA++	L-15	144	100	3107 ± 475	0.89 ± 0.12	757 ± 238 (100)	-	-
IB	L-15	0	58	3247 ± 391	1.00 ± 0.00	382 ± 52 (100)	-	-
IB	L-15	144	58	2499 ± 135	0.97 ± 0.03	579 ± 1.3 (100)	-	-
IB+	L-15	0	100	5190 ± 518	0.57 ± 0.09	727 ± 161 (100)	-	-
IB+	L-15	144	100	3586 ± 471	1.00 ± 0.00	216 ± 44 (100)	-	-
IA	Aquarium H_2_O	0	67.6	2929 ± 440	1.00 ± 0.00	265 ± 82 (100)	-	−28.3 ± 0.7
IA	Aquarium H_2_O	48	67.6	2906 ± 239	1.00 ± 0.00	350 ± 49 (100)	-	-
IA	Aquarium H_2_O	96	67.6	4401 ± 653	0.99 ± 0.01	486 ± 234 (100)	-	−21.3 ± 0.2
IB	Aquarium H_2_O	0	67.6	1900 ± 134	1.00 ± 0.00	507 ± 45 (98.2)	118 (1.8)	−28.5 ± 0.8
IB	Aquarium H_2_O	48	67.6	2577 ± 218	1.00 ± 0.00	500 ± 54 (98)	241 (2)	-
IB	Aquarium H_2_O	96	67.6	3574 ± 349	0.99 ± 0.01	517 ± 29 (100)	-	−25.6 ± 0.6

Dynamic light scattering analysis was performed in Milli-Q water, L-15 culture medium, and aquarium water. ^a^ Z-average size (Z-ave), ^b^ polydispersity index (PDI), ^c^ average hydrodynamic diameter (HDD), ^d^ relative intensities of size peak (%). Data are represented as the mean ± SEM (n ≥ 4).

## Data Availability

The data presented in this study are available upon request from the corresponding author. The data are not publicly available due to limitations on the availability of data until the end of the project. Thereafter, data presented in this study will be openly available at https://digital.csic.es, accessed on 17 December 2023.
